# MicroRNAs in Autoimmune Diseases

**DOI:** 10.1155/2014/527895

**Published:** 2014-06-04

**Authors:** Zigang Qu, Wenhui Li, Baoquan Fu

**Affiliations:** State Key Laboratory of Veterinary Etiological Biology, Key Laboratory of Veterinary Public Health of the Ministry of Agriculture, Key Laboratory of Veterinary Parasitology of Gansu Province, Lanzhou Veterinary Research Institute, Chinese Academy of Agricultural Sciences, Lanzhou, Gansu 730046, China

## Abstract

Autoimmune diseases (ADs) are featured by body's immune responses being directed towards its own specific target organs or multiple organ systems, causing persistent inflammation and consequent tissue damage. miRNAs are small noncoding RNAs in a size of approximately 22 nt that play important regulatory roles in many organisms by cleavage or translational inhibition of targeted mRNAs. Many miRNAs are reported to be differentially expressed in ADs and may play a pivotal role in regulating immune responses and autoimmunity. In this review, current research progress in the miRNAs in ADs was elucidated.

## 1. Introduction

MicroRNAs (miRNAs) are small noncoding regulatory RNAs that are involved in regulation of gene expression in a posttranscriptional manner [1]. The first miRNA,* lin-*4, was discovered in* C. elegans* in 1993 [2] and now it is known that miRNAs are evolutionarily conserved across diverse phyla, from nematodes to humans [3]. The number of miRNAs in the human genome is more than 1000 which regulate over 30% of the total number of human genes [4]. Most miRNAs are derived from self-reliant miRNA genes or introns of genes coding for proteins and most of them are transcribed via RNA polymerase II to generate pri-miRNAs. After pri-miRNAs were initially processed by Drosha and DGCR8 that are located in the nucleus, the resulted miRNA precursors, pre-miRNAs, are delivered to the cytoplasm where the miRNA hairpin structure is processed by Dicer enzyme, resulting in a miRNA double complex. One of the RNA strands is then loaded into small RNA-induced silencing complex (RISC) and subsequently directs this complex to the 3′ untranslated regions (UTRs) of target mRNAs, inducing the repression of target protein expression [5]. miRNAs participated in many physiological processes and can regulate cellular processes such as differentiation, proliferation, and apoptosis [6, 7]. Recently, miRNAs have been revealed to play a significant role in autoimmune processes and autoimmune diseases (ADs) such as systemic lupus erythematosus (SLE) and rheumatoid arthritis (RA) [8, 9].

## 2. miRNAs in ADs

ADs are prolonged disease conditions originating from the deficiency of immunological tolerance to autoantigens and consequent pathological status that inflict exclusive target organs or multiple organ systems [10]. The prevalence of ADs in world population is more than 3% and 80% of ADs patients are women [11]. Changes in the expression of several miRNAs have been uncovered in ADs, for instance, rheumatoid arthritis (RA), type 1 diabetes mellitus (T1DM), multiple sclerosis (MS), Sjögren's syndrome (SS), systemic lupus erythematosus (SLE), inflammatory bowel disease (IBD), psoriasis (PS), primary biliary cirrhosis (PBC), and idiopathic thrombocytopenic purpura (ITP). The roles of miRNAs in these disorders are discussed below and summarized in Table 1.

## 3. Rheumatoid Arthritis (RA)

Rheumatoid arthritis (RA) is a systemic autoimmune abnormality mainly featured by the inflammation of synovial tissue that can cause bone and cartilage destruction [12]. Several studies indicated that miR-146a and miR-155 were persistently upregulated in peripheral blood mononuclear cells (PBMCs) [13], synovial fibroblasts (RASFs) [14], synovial fluid [15], PBMC-derived CD4^+^ T-cells [16], and Th-17 cells from patients with RA versus those of healthy controls or osteoarthritis patients (OA) [17].

Although impaired apoptosis of synovial fibroblasts is critical for the pathogenesis of RA, the miRNA-dependent regulation of apoptosis is rarely known. Recent research revealed that both miR-34a and miR-34a* are related with the regulation of apoptotic pathways. The demethylation of the miR-34a* promoter evidently enhanced the expression levels of miR-34a*. miR-34a* promotes apoptosis in both FasL- and TRAIL-stimulated RA synovial fibroblasts (RASFs), whereas the overexpression of the mature strand miR-34a shelters cells from FasL-mediated apoptosis but has no effect on TRAIL-induced cell death [18]. miR-124 is another RA-related miRNA that is involved in cell proliferation. Cyclin-dependent kinase 2 (CDK2) and monocyte protein-1 (MCP) are two targets of miR-124 that are tightly regulated. Thus, miR-124 may be a critical regulator of the synovial inflammatory milieu in RA [19]. Upregulation of miR-346 in RA fibroblast-like synoviocytes was also reported [20], and it was shown that miR-346 could indirectly regulate IL-18 release. miR-203 was upregulated in synovial fibroblasts, in patients with RA, and elevated levels of miR-203 lead to enhanced secretion of MMP-1 and IL-6 via the NF-*κ*B pathway and consequently lead to the activated phenotype of synovial fibroblasts in RA, thus; revealing that miR-203 is a proinflammatory factor in RA [21].

In another experiment, miR-146a expression was remarkably higher while the expression of miR-363 and miR-498 was lower [16], Let-7a was found to be downregulated in PBMCs from RA patients [13], whereas miR-132 and miR-16 were upregulated. Upregulated miRNA-155 expression in PBMCs and fibroblast-like synoviocytes in RA patients was shown to be protective against the inflammatory effects, partially due to the ability of miRNA-155 to attenuate IKBKE expression [22]. The plasma concentrations of miR-24 and miR-125a-5p were potential diagnostic markers of RA [23]. Another potential diagnostic biomarker, miR-140, was downregulated in human articular chondrocytes, suggesting its involvement in regulatory pathways that control cartilage development and modulate the IL-1 response [24]. The expression of miRNA-323-3p, which is located on chromosome 14q32.31, was found to be upregulated in synovial fibroblasts and can serve as a biomarker for immune and inflammatory responses as well as the enhancement of Wnt/cadherin pathway activation [25].

## 4. Type 1 Diabetes Mellitus (T1DM)

Diabetes mellitus (DM) is an intricate, multiple organ system disease that represents the most typical metabolic disturbance [26, 27] and comprises T1DM and Type 2 DM. T1DM originates from insulin deficiency [28], which is consequent to the T cell mediated destruction of *β*-cells that produce insulin in human pancreas islets. Several miRNAs are associated with T1DM, including miR-375. miR-375 knockout mice were hyperglycemic and glucose intolerant while pancreatic *β*-cell mass was decreased because of impaired proliferation [29], which revealed the important role of miR-375 in normal glucose metabolism, *α*- and *β*-cell turnover, and adaptive *β*-cell growth in response to increasing insulin demand during insulin resistance. Thus, miR-375 expression may have an indirect influence on T1DM. Key circulating miRNAs that predict progressive *β*-cell destruction and regeneration in children with newly diagnosed T1DM were identified [30]. Twelve miRNAs were upregulated and some of them were associated with apoptosis and *β*-cell networks. In particular, a “tissue-specific” miR-25 involved in glycemic controls 3 months after diagnosis in new onset T1D children may be a predictive biomarker for tissue physiopathology and potential targets for clinical therapy.

## 5. Multiple Sclerosis (MS)

MS (OMIM 126200) is an autoimmune disease of the central nervous system that is caused by inflammatory and neurodegenerative processes interactions and typically leads to intermittent neurological disorder followed by progressive accumulation of disability [31]. Studies have recently revealed the involvement of miRNAs in MS with miRNA profiling techniques [32–39].

Investigations have shown that miRNAs play an important role in Th-17 polarization and the pathological mechanism of MS. The expression of a Th-17 cell-related miRNA, miR-326, was highly relevant to disease severity in MS patients and mice with experimental autoimmune encephalomyelitis (EAE).* In vivo* silencing of miR-326 caused a decrease in the number of Th-17 cells and mild EAE, whereas its overexpression contributed to an expansion in the number of Th-17 cells and severe EAE. miR-326 could promote Th-17 differentiation through targeting Ets-1, a negative regulator of Th-17 differentiation [32].

Three miRNAs (miR-18b, miR-599, and miR-493) were significantly upregulated in relapsing-remitting MS (RRMS) patients compared with healthy volunteers [33]. By using microarray technology, differential miRNA expression in whole blood samples of RRMS patients was studied and found that ten miRNAs were significantly dysregulated [34]. Furthermore, miRNAs in MS lesions were assayed and three miRNAs were expressed higher in active versus inactive MS lesions. These miRNAs were found to target the 3′ UTR of CD47 gene [35].

miRNA expression profiles in serum samples from RRMS, SPMS, and PPMS patients and healthy controls were analyzed and it was revealed that miR-21 and miR-106b were upregulated in all types of MS and miR-106b falls into the miR-17–92 cluster category [36]. Furthermore, members of the miR-17–92 cluster were downregulated in the B-lymphocytes of MS patients [37]. When compared with healthy controls, 23 miRNAs were differentially expressed in the CD4(+) CD25(+high) T regulatory cells obtained from RRMS patients in stable condition [38]. Altogether 365 miRNAs in the lymphocytes of RRMS patients were investigated and found that miR-17-5p, which is associated with autoimmunity, was upregulated in the CD4^+^ cells of MS patients [39].

## 6. Sjögren's Syndrome (SS)

SS is considered to be an AD featured by prolonged inflammation involving the exocrine glands [40]. Most SS patients have a slowly progressive course, and many do not need intensive immunosuppression. Therefore, SS is an important model for studying many aspects of miRNAs in a systemic AD. Microarrays assay conducted in the minor salivary glands (MCGs) of healthy controls indicated that miRNA expression profiles can be available to distinguish the glands of SS patients from those of controls and also to distinguish subsets of SS patients with low- or high-grade inflammation. Additionally, the miR-17-92 cluster profile of patients, which has been involved in specific types of lymphocytes and lymphocytic disorder, was downregulated [41]. In another study, miR-146 was upregulated in the SS patients and the PBMCs and the salivary glands of SS-prone mice at 8 and 20 weeks of age. miR-146 may increase phagocytic activity and suppress inflammatory cytokine production [42]. Taken together, the above data reveals that miR-146 may be used as a marker of the initiation and progression of SS.

## 7. Systemic Lupus Erythematosus (SLE)

Systemic lupus erythematosus (SLE) is a prolonged autoimmune disease with intricate etiology and multiple clinical symptoms [43]. Microarray analysis of miRNA expression in peripheral blood cells of SLE patients revealed that 16 miRNAs were differentially expressed in SLE [44]. miR-146a was identified as a negative modulator of natural immunity and low miR-146a expression was negatively correlated with clinical disease manifestation in SLE patients. miR-146a directly inhibited the downstream transactivation of type I IFN at the molecular level and targeted IFN regulatory factor 5 and STAT-1 [45]. The relationship between promoter variant of miR-146a and SLE was confirmed, in which the risk allele showed lower binding ability to Ets-1, consequently leading to downregulated levels of miR-146a in SLE patients [46]. The TLR7-TLR8 region was mapped and demonstrated that the functional SNP rs3853839 was most likely associated with SLE in three non-Asian ancestry populations [47]. miR-21 is involved in SLE, with a role in the T-cell response through the regulation of programmed cell death (PDCD4) [48]. Expression levels of miR-146a (and miR-155) were observed to be downregulated in serum from SLE patients [49]. Another study indicated that hsa-miR-371-5P, hsa-miR-1224-3P, and hsa-miR-423-5P were involved in LN [50]. The role of miRNAs in epigenetic processes is also being investigated. As key miRNAs in SLE, miR-148a and miR-21 play roles in DNA hypomethylation in the disease state [51]. Additionally, recent study demonstrated that miRNA-126 leads to SLE by targeting DNA methylation [52]. One study demonstrated that miR-15 was upregulated in spleen cells and plasma in a SLE murine model, and the involvement of increasing miR-15a in AD development in B/W mice suggested that the downregulation of his miRNA might be a useful therapeutic option. Thus, miR-15 may participate in the pathogenesis of lupus [53].

## 8. Inflammatory Bowel Disease (IBD)

IBD is a subset of inflammatory conditions that is involved in the colon and small intestine [54]. Crohn's disease (CD) and ulcerative colitis (UC) are the two major types of chronic idiopathic IBD. Although UC and CD have distinct clinical conditions with distinguishing clinical and histological manifestation, a gold standard for diagnostic remains a mystery [55]. It is suggested that a better understanding of the complex etiology and immunological principle that trigger the development of UC and CD will result in improved therapy for IBD.

miRNAs profile in the blood of active CD patients and active UC patients was different from that of healthy control. Five miRNAs were significantly upregulated and two miRNAs (149* and miRplus-F1065) were downregulated in CD patients compared to healthy volunteers, while twelve miRNAs were significantly upregulated and miRNA-505* was significantly downregulated in patients of active UCs. Comparison of expression levels in active UC and CD patients indicated ten miRNAs were upregulated while one miRNA was significantly downregulated in the blood of active UC patients [56]. In another study, 11 CD-associated serum miRNAs were identified, including miR-195, miR-16, miR-93, miR-140, miR-30e, miR-20a, miR-106a, miR-192, miR-21, miR-484, and let-7b [57]. Moreover, in CD patients, eleven miRNAs were upregulated compared to healthy controls. However, in the UC patients, several miRNAs were increased remarkably [58].

By analyzing expression level with microarray technique, 31 differentially expressed platelet-derived miRNAs have been confirmed and were regarded as promising biomarkers for noninvasive prediction of UC cases, ultimately revealing that miR-188-5p, miR-422a, miR-378, miR-500, miR-501-5p, miR-769-5p, and miR-874 were dysregulated [59]. Differentially expressed miRNAs in sigmoid colon biopsies of active UC patients versus healthy controls were assayed [60, 61] and in another study miR-150 was significantly upregulated in the inflamed colonic mucosa of UC patients versus healthy volunteers [62].

The expression profiles of 467 miRNAs in patients with active terminal ileal CD compared to control tissues were assayed and the result showed four miRNAs were significantly increased in active ileal CD tissue [60]. A similar study analyzed more than 300 miRNAs in colonic tissue samples of UC and CD patients and identified a set of eight miRNAs that could differentiate quiescent IBD from controls and a distinct category of 15 miRNAs that could differentiate between quiescent UC and CD [63]. Thus, these data suggest miRNA can be used as diagnostic markers and as a factor that participated in IBD pathogenesis. The interaction between certain specific miRNAs and their target genes was also studied. For example miR-150 played a role in targeting c-Myb [62] and a negative correlation between miR-143 and its target genes K-RAS, API-5, and MEK-2 and between miR-145 and its target gene IRS-1 was discovered [64], while another study demonstrated miR-7 can target CD98 which should interfere with the natural proliferation and differentiation of enterocytes [65]. Single miRNAs and their relationships to single nucleotide polymorphisms (SNPs) are also being studied. miR-196 was significantly expressed in intestinal epithelial cells within inflamed CD when compared with controls. These authors revealed a negative correlation between miR-196 and a protective variant of the immunity-related GTPase M (IRGM c.313C) in inflammatory conditions [66]. Taken together, these studies illustrate the potential use of miRNAs as predictive biomarkers and highlight the potential of miRNA profile-based diagnostic tools and miRNA-based inhibitors.

## 9. Psoriasis (PS)

PS is a prolonged inflammatory immune-mediated skin disturbance and is suggested to be initiated by an assemblage of genetic and immunological abnormalities [67]. Several miRNAs are suggested to be associated with PS pathogenesis through their modulation of protein expression and cellular functions. miR-203 was expressed significantly higher in PS skin versus normal controls and could inhibit SOCS-3, which lead to the prolonged activation of STAT3 and immune cell infiltration [68]. Another upregulated miR-146 is associated with the modulation of innate immune responses and TNF-pathway [69]. miR-492 could suppress the expression of BSG and the BSG rs8259 polymorphism is involved in decreased PS susceptibility due to its effects on miR-492 binding [70].

## 10. Primary Biliary Cirrhosis (PBC)

PBC is a constant liver disease featured by damage of intrahepatic bile-duct and cirrhosis and the appearance of highly specific antimitochondrial antibodies and autoreactive T-cells indicated its autoimmune nature [71]. It was found that expression levels of three miRNAs (miR-299-5p, miR-328, and miR-371) were upregulated whereas the expression levels of three miRNAs (miR-26a, miR-122a, and miR-99) were downregulated when comparing the terminal stages of PBC in patients with those of normal controls, thus suggesting that these miRNAs may be used as diagnostic biomarkers [72].

## 11. Idiopathic Thrombocytopenic Purpura (ITP)

ITP is an autoimmune disease characterized by a low platelet count, and production of autoantibodies is one of the primary causes of this disease [73]. Patients produce antibodies to specific glycoproteins within platelet membrane, leading to severe destruction of peripheral platelet. In peripheral blood cells that are derived from ITP patients, five miRNAs were upregulated while fourteen miRNAs were upregulated, while fourteen miRNAs were significantly downregulated [44]. Further researches should be carried out to uncover the interactions between these miRNAs and their targets in ITP.

## 12. Conclusion

miRNAs have a versatile range of abilities to manipulate posttranscriptional mechanisms leading to control of gene expression. The complex nature of miRNAs regulatory interactions with the other pathways (autophagy, apoptosis, and inflammatory pathways) should be further investigated to identify regulated targets and elaborate the dominant miRNAs factors engaged in the pathogenesis of ADs.

## Figures and Tables

**Table 1:** miRNAs in autoimmune disease.

Autoimmune disease	miRNA	Expression localization and regulatory role	Documented and postulated effect	Reference
Rheumatoid arthritis (RA)	miR-146a 155	PMBC ↑	P	[13]
	miR-146a 155	Synovial tissue RASFs and synovial fluid ↑	P	[14, 15]
	miR-146a 155	PMBC-derived CD4^+^ T cell ↑	P	[16]
	miR-146a 155	Th-17 cell ↑	P	[17]
	miR-124	↑ cell proliferation MCP-1 production	Inflammatory pathogenesis	[19]
	miR-34*	Demethylated promoter increases miR-34a* expression	Promotes apoptosis	[18]
	miR-346	Regulates IL-18 release	Inflammatory pathogenesis	[20]
	miR-203a	MMP IL-6 production ↑	Proinflammatory factor	[21]
	miR-363 498	Plasma ↓	P	[16]
	miR-24 125a-5p	Plasma ↑	P	[23]
	Let-7a	PMBC ↓	P	[13]
	miR-132 16	PMBC ↑	P	[13]
	miR-140	Human articular chondrocytes ↓	Regulates pathways that control cartilage development and homeostasis	[24]
	miR-323-3p	Synovial fibroblast ↑	Biomarker for immune and inflammatory response enhances Wnt/cadherin pathway activation	[25]
	miR-155	PBMC and fibroblast-like synoviocytes ↑	Against inflammatory effect	[22]

Multiple sclerosis (MS)	miR-18b 599 493	RRMS ↑	P	[33]
	miR-145 186 664 20b 422a 142-3p 584 223 1275 491-5p	Dysregulated in whole blood	P	[34]
	miR-34a 155 326	↑ active compared to inactive MS	P Targets the 3′ UTR of CD47	[35]
	miR-21 106b	RRMS SPMS PPMS serum ↑	P	[36]
	miR-17-5p, 19a/b, 20a and 92b	B-lymphocytes of MS patients ↓	P	[37]
	Several miRNAs	Differentially expressed in CD4^+^ CD25^high^ cells	P	[38]
	miR-17-5p	CD4^+^ cell from RRMS patients ↑	P	[39]
	miR-326	MS patients ↑	Promotes Th-17 differentiation and pathogenesis	[32]

Systemic lupus erythematosus (SLE)	Several miRNAs	Differentially expressed in lupus nephritis and SLE	P	[44]
	miR-146a	Target IFN regulatory factor 5 and STAT-1 repress the transactivation of type I IFN miR146 promoter variant decreased binding to transcription factor Ets-1 causing reduced miR-146a level in SLE patients	Negative regulator of innate immunity correlated with inflammatory pathway	[45, 46]
	miR-3148	Binds with SNP rs3853839 and TLR7-TLT8 regions	Distinguishes populations of non-Asians and Asians	[47]
	miR-21	Regulating PDCD	Immune response	[48]
	miR-146a	Serum of SLE patients ↓	P	[49]
	miR-155	Serum of SLE patients ↓	P	[49]
	Hsa-miR-371-5P 1224-3P 423-5P	Differentially expressed in lupus nephritis	P	[50]
	miR-15	Splenic cellular and plasma in murine model ↑	Pathogenesis	[53]
	miR-148a 21	DNA methylation in lupus	Epigenetic regulation	[51]

Inflammatory bowel disease (IBD)	miRs-199a-5p 362-3p 532-3p plus-E1271 340*	Peripheral blood from active CD patients ↑	P	[56]
	miR-149* plus-F1065	Peripheral blood from active CD patients ↓	P	[56]
	Several miRNAs	Blood from active UC patients ↑	P	[56]
	miR-505*	Blood from active UC patients ↓	P	[56]
	miRs-28-5p 103-2* 1495*151-5p 340* 505* 532-3p plus-E1153	↑ peripheral blood of active UC patients versus active CD patients	P	[56]
	miR-505*	↓ in peripheral blood of active UC patients versus active CD patient	P	[56]
	miRs-195 16 93 140 30e 20a 106a 192 21 484 let-7b	Differentially expressed in serum from CD patients	P	[57]
	miRs-16 23a 29a 106a 107 126 191 199a-5p 200c 362-3p 532-3p	Blood from CD patients ↑	P	[58]
	miRs-16 21 28-5p 151-5p 155 199a-5p	Blood from UC patients ↑	P	[58]
	miRs-188-5p 422a 378 500 501-5p 769-5p 874	Dysregulated in microvesicles peripheral blood mononuclear cells' platelets of UC patients	P	[59]
	miRs-192 375 422b 16 21 23a 24 29a 126 195	Dysregulated in sigmoid colon biopsies from active UC patients	P	[60]
	miR-21 155	Dysregulated in inflamed colonic mucosa from active UC patients		[61]
	miR-150	Inflamed colonic mucosa of UC patients ↑	P Targets c-Myb	[62]
	miRs-26a 29a 29b 30c 126* 127-3p 196a 324-3p	Dysregulated in colonic tissue of IBD	P	[63]
	Several miRNAs	Differentially expressed in colonic tissue from quiescent UC versus CD patients	P	[63]
	miR-143	Negatively correlates with *MEK-2 *	Unknown	[64]
	miR-145	Negatively correlates with *IRS-1 K-RAS API-5 *	Unknown	[64]
	miR-7	Negatively correlates with its target-CD98	Interferes with natural proliferation and differentiation of enterocytes	[65]

“↑” represents upregulated.

“↓” represents downregulated.

“P” represents potential diagnostic biomarker.

## References

[1] Bartel DP (2004). MicroRNAs: genomics, biogenesis, mechanism, and function. *Cell*.

[2] Lee RC, Feinbaum RL, Ambros V (1993). The *C. elegans* heterochronic gene lin-4 encodes small RNAs with antisense complementarity to lin-14. *Cell*.

[3] Farh KKH, Grimson A, Jan C (2005). The widespread impact of mammalian microRNAs on mRNA repression and evolution. *Science*.

[4] Perera RJ, Ray A (2007). MicroRNAs in the search for understanding human diseases. *BioDrugs*.

[5] Eulalio A, Huntzinger E, Izaurralde E (2008). Getting to the root of miRNA-mediated gene silencing. *Cell*.

[6] Ambros V (2003). MicroRNA pathways in flies and worms: growth, death, fat, stress, and timing. *Cell*.

[7] Ambros V (2004). The functions of animal microRNAs. *Nature*.

[8] Zhou X, Jeker LT, Fife BT (2008). Selective miRNA disruption in T reg cells leads to uncontrolled autoimmunity. *Journal of Experimental Medicine*.

[9] Pauley KM, Cha S, Chan EKL (2009). MicroRNA in autoimmunity and autoimmune diseases. *Journal of Autoimmunity*.

[10] Anaya JM, Shoenfeld Y, Correa PA, García-Carrasco M, Cervera R (2005). *Autoinmunidad y Enfermedad Autoinmune*.

[11] Cooper GS, Stroehla BC (2003). The epidemiology of autoimmune diseases. *Autoimmunity Reviews*.

[12] Klareskog L, Catrina AI, Paget S (2009). Rheumatoid arthritis. *The Lancet*.

[13] Pauley KM, Satoh M, Chan AL, Bubb MR, Reeves WH, Chan EKL (2008). Upregulated miR-146a expression in peripheral blood mononuclear cells from rheumatoid arthritis patients. *Arthritis Research and Therapy*.

[14] Stanczyk J, Leslie Pedrioli DM, Brentano F (2008). Altered expression of microRNA in synovial fibroblasts and synovial tissue in rheumatoid arthritis. *Arthritis and Rheumatism*.

[15] Murata K, Yoshitomi H, Tanida S (2010). Plasma and synovial fluid microRNAs as potential biomarkers of rheumatoid arthritis and osteoarthritis. *Arthritis Research and Therapy*.

[16] Li J, Wan Y, Guo Q (2010). Altered microRNA expression profile with miR-146a upregulation in CD4^+^ T cells from patients with rheumatoid arthritis. *Arthritis Research and Therapy*.

[17] Niimoto T, Nakasa T, Ishikawa M (2010). MicroRNA-146a expresses in interleukin-17 producing T cells in rheumatoid arthritis patients. *BMC Musculoskeletal Disorders*.

[18] Niederer F, Trenkmann M, Ospelt C (2012). Down-regulation of microRNA-34a∗ in rheumatoid arthritis synovial fibroblasts promotes apoptosis resistance. *Arthritis & Rheumatology*.

[19] Nakamachi Y, Kawano S, Takenokuchi M (2009). MicroRNA-124a is a key regulator of proliferation and monocyte chemoattractant protein 1 secretion in fibroblast-like synoviocytes from patients with rheumatoid arthritis. *Arthritis and Rheumatism*.

[20] Alsaleh G, Suffert G, Semaan N (2009). Bruton’s tyrosine kinase is involved in miR-346-related regulation of IL-18 release by lipopolysaccharide-activated rheumatoid fibroblast-like synoviocytes. *Journal of Immunology*.

[21] Stanczyk J, Ospelt C, Karouzakis E (2011). Altered expression of microRNA-203 in rheumatoid arthritis synovial fibroblasts and its role in fibroblast activation. *Arthritis and Rheumatism*.

[22] Long L, Yu P, Liu Y (2013). Upregulated microRNA-155 expression in peripheral blood mononuclear cells and fibroblast-like synoviocytes in rheumatoid arthritis. *Clinical and Developmental Immunology*.

[23] Murata K, Furu M, Yoshitomi H (2013). Comprehensive microRNA analysis identifies miR-24 and miR-125a-5p as plasma biomarkers for rheumatoid arthritis. *PLoS ONE*.

[24] Miyaki S, Nakasa T, Otsuki S (2009). MicroRNA-140 is expressed in differentiated human articular chondrocytes and modulates interleukin-1 responses. *Arthritis and Rheumatism*.

[25] Xu T, Huang C, Chen Z, Li J (2013). MicroRNA-323-3p: a new biomarker and potential therapeutic target for rheumatoid arthritis. *Rheumatology International*.

[26] Adams KF, Schatzkin A, Harris TB (2006). Overweight, obesity, and mortality in a large prospective cohort of persons 50 to 71 years old. *New England Journal of Medicine*.

[27] Shaw JE, Sicree RA, Zimmet PZ (2010). Global estimates of the prevalence of diabetes for 2010 and 2030. *Diabetes Research and Clinical Practice*.

[28] Ilonen J, Akerblom HK (1999). New technologies and genetics of type 1 diabetes. *Diabetes Technology & Therapeutics*.

[29] Poy MN, Hausser J, Trajkovski M (2009). miR-375 maintains normal pancreatic *α*- and *β*-cell mass. *Proceedings of the National Academy of Sciences of the United States of America*.

[30] Nielsen LB, Wang C, Sørensen K (2012). Circulating levels of microRNA from children with newly diagnosed type 1 diabetes and healthy controls: evidence that miR-25 associates to residual beta-cell function and glycaemic control during disease progression. *Experimental Diabetes Research*.

[31] Compston A, Coles A (2008). Multiple sclerosis. *The Lancet*.

[32] Du C, Liu C, Kang J (2009). MicroRNA miR-326 regulates TH-17 differentiation and is associated with the pathogenesis of multiple sclerosis. *Nature Immunology*.

[33] Otaegui D, Baranzini SE, Armañanzas R (2009). Differential micro RNA expression in PBMC from multiple sclerosis patients. *PLoS ONE*.

[34] Keller A, Leidinger P, Lange J (2009). Multiple sclerosis: microRNA expression profiles accurately differentiate patients with relapsing-remitting disease from healthy controls. *PLoS ONE*.

[35] Junker A, Krumbholz M, Eisele S (2009). MicroRNA profiling of multiple sclerosis lesions identifies modulators of the regulatory protein CD47. *Brain*.

[36] Lindberg RLP, Hoffmann F, Kuhle J, Kappos L (2010). Circulating microRNAs as indicators for disease course of multiple sclerosis. *Multiple Sclerosis*.

[37] Sievers C, Hoffmann F, Fontoura P, Kappos L, Lindberg RLP (2010). Effect of natalizumab on microRNA expression in B-lymphocytes of relapsing-remitting multiple sclerosis patients. *Multiple Sclerosis*.

[38] de Santis G, Ferracin M, Biondani A (2010). Altered miRNA expression in T regulatory cells in course of multiple sclerosis. *Journal of Neuroimmunology*.

[39] Lindberg RLP, Hoffmann F, Mehling M, Kuhle J, Kappos L (2010). Altered expression of miR-17-5p in CD4^+^ lymphocytes of relapsing-remitting multiple sclerosis patients. *European Journal of Immunology*.

[40] Nikolov NP, Illei GG (2009). Pathogenesis of Sjögren's syndrome. *Current Opinion in Rheumatology*.

[41] Alevizos I, Bajracharya SD, Alexander S, Turner RJ, Illei GG (2009). MicroRNA profiling of minor salivary glands identifies disease and inflammation biomarkers in Sjogren's syndrome patients. *Arthritis & Rheumatism*.

[42] Pauley KM, Stewart CM, Gauna AE (2011). Altered miR-146a expression in Sjögren’s syndrome and its functional role in innate immunity. *European Journal of Immunology*.

[43] D’Cruz DP, Khamashta MA, Hughes GR (2007). Systemic lupus erythematosus. *The Lancet*.

[44] Dai Y, Huang YS, Tang M (2007). Microarray analysis of microRNA expression in peripheral blood cells of systemic lupus erythematosus patients. *Lupus*.

[45] Tang Y, Luo X, Cui H (2009). MicroRNA-146a contributes to abnormal activation of the type I interferon pathway in human lupus by targeting the key signaling proteins. *Arthritis and Rheumatism*.

[46] Luo X, Yang W, Ye DQ (2011). A functional variant in microRNA-146a promoter modulates its expression and confers disease risk for systemic lupus erythematosus. *PLoS Genetics*.

[47] Deng Y, Zhao J, Sakurai D (2013). MicroRNA-3148 modulates allelic expression of toll-like receptor 7 variant associated with systemic lupus erythematosus. *PLoS Genetics*.

[48] Stagakis E, Bertsias G, Verginis P (2011). Identification of novel microRNA signatures linked to human lupus disease activity and pathogenesis: MiR-21 regulates aberrant T cell responses through regulation of PDCD4 expression. *Annals of the Rheumatic Diseases*.

[49] Wang G, Tam LS, Li EKM (2010). Serum and urinary cell-free MiR-146a and MiR-155 in patients with systemic lupus erythematosus. *Journal of Rheumatology*.

[50] Te JL, Dozmorov IM, Guthridge JM (2010). Identification of unique microRNA signature associated with lupus nephritis. *PLoS ONE*.

[51] Pan W, Zhu S, Yuan M (2010). MicroRNA-21 and microRNA-148a contribute to DNA hypomethylation in lupus CD4^+^ T cells by directly and indirectly targeting DNA methyltransferase 1. *Journal of Immunology*.

[52] Zhao S, Wang Y, Liang Y (2011). MicroRNA-126 regulates DNA methylation in CD4^+^ T cells and contributes to systemic lupus erythematosus by targeting DNA methyltransferase 1. *Arthritis and Rheumatism*.

[53] Yuan Y, Kasar S, Underbayev C (2012). Role of microRNA-15a in autoantibody production in interferon-augmented murine model of lupus. *Molecular Immunology*.

[54] Hanauer SB (1996). Inflammatory bowel disease. *New England Journal of Medicine*.

[55] Sands BE (2004). From symptom to diagnosis: clinical distinctions among various forms of intestinal inflammation. *Gastroenterology*.

[56] Wu F, Guo NJ, Tian H (2011). Peripheral blood MicroRNAs distinguish active ulcerative colitis and Crohn’s disease. *Inflammatory Bowel Diseases*.

[57] Zahm AM, Thayu M, Hand NJ, Horner A, Leonard MB, Friedman JR (2011). Circulating microRNA is a biomarker of pediatric crohn disease. *Journal of Pediatric Gastroenterology and Nutrition*.

[58] Paraskevi A, Theodoropoulos G, Papaconstantinou I, Mantzaris G, Nikiteas N, Gazouli M (2012). Circulating MicroRNA in inflammatory bowel disease. *Journal of Crohn’s and Colitis*.

[59] Duttagupta R, DiRienzo S, Jiang R (2012). Genome-wide maps of circulating miRNA biomarkers for Ulcerative Colitis. *PLoS ONE*.

[60] Wu F, Zhang S, Dassopoulos T (2010). Identification of microRNAs associated with ileal and colonic Crohn’s disease. *Inflammatory Bowel Diseases*.

[61] Takagi T, Naito Y, Mizushima K (2010). Increased expression of microRNA in the inflamed colonic mucosa of patients with active ulcerative colitis. *Journal of Gastroenterology and Hepatology*.

[62] Bian Z, Li L, Cui J (2011). Role of miR-150-targeting c-Myb in colonic epithelial disruption during dextran sulphate sodium-induced murine experimental colitis and human ulcerative colitis. *Journal of Pathology*.

[63] Fasseu M, Tréton X, Guichard C (2010). Identification of restricted subsets of mature microRNA abnormally expressed in inactive colonic mucosa of patients with inflammatory bowel disease. *PLoS ONE*.

[64] Pekow JR, Dougherty U, Mustafi R (2012). MiR-143 and miR-145 are downregulated in ulcerative colitis: putative regulators of inflammation and protooncogenes. *Inflammatory Bowel Diseases*.

[65] Nguyen HTT, Dalmasso G, Yan Y (2010). MicroRNA-7 modulates CD98 expression during intestinal epithelial cell differentiation. *Journal of Biological Chemistry*.

[66] Brest P, Lapaquette P, Souidi M (2011). A synonymous variant in IRGM alters a binding site for miR-196 and causes deregulation of IRGM-dependent xenophagy in Crohn’s disease. *Nature Genetics*.

[67] Griffiths CE, Barker JN (2007). Pathogenesis and clinical features of psoriasis. *The Lancet*.

[68] Sonkoly E, Wei T, Janson PCJ (2007). MicroRNAs: novel regulators involved in the pathogenesis of psoriasis?. *PLoS ONE*.

[69] Sonkoly E, Ståhle M, Pivarcsi A (2008). MicroRNAs: novel regulators in skin inflammation. *Clinical and Experimental Dermatology*.

[70] Wu LS, Li FF, Sun LD (2011). A miRNA-492 binding-site polymorphism in BSG (basigin) confers risk to psoriasis in Central South Chinese population. *Human Genetics*.

[71] Selmi C, Bowlus CL, Gershwin ME, Coppel RL (2011). Primary biliary cirrhosis. *The Lancet*.

[72] Padgett KA, Lan RY, Leung PC (2009). Primary biliary cirrhosis is associated with altered hepatic microRNA expression. *Journal of Autoimmunity*.

[73] Nurden AT, Viallard JF, Nurden P (2009). New-generation drugs that stimulate platelet production in chronic immune thrombocytopenic purpura. *The Lancet*.

